# Effects of radiofrequency ablation versus other ablating techniques on hepatocellular carcinomas: a systematic review and meta-analysis

**DOI:** 10.1186/s12957-017-1196-2

**Published:** 2017-07-10

**Authors:** Wen Luo, Yunfei Zhang, Guangbin He, Ming Yu, Minjuan Zheng, Liwen Liu, Xiaodong Zhou

**Affiliations:** 1 Department of Ultrasound, Xijing Hospital, Fourth Military Medical University, No. 127 Changle Xi Road, Xi’an, China; 2 Research Institution of Bone tumor, Tangdu Hospital, Fourth Military Medical University, Xi’an, China

**Keywords:** Hepatocellular carcinoma, Ablative techniques, Radiofrequency ablation, Therapeutic effects

## Abstract

**Background:**

Percutaneous ablation has quickly arisen as one of the important alternative treatments for hepatocellular carcinoma (HCC). We aimed to compare the therapeutic effects of radiofrequency ablation (RFA) and other ablative techniques on HCCs.

**Methods:**

Databases were searched to identify literature on complete tumor ablation (CTA), overall survival (OS), local tumor recurrence (LTR), and complications of RFA in the treatment of HCC, compared with those of microwave ablation (MWA), percutaneous ethanol injection (PEI), PEI plus RFA, cryoablation (CRA), laser ablation (LSA), and high-intensity focused ultrasound. Randomized controlled trials and high-quality cohort studies were included in the assessment.

**Results:**

The effects of MWA and CRA appeared to be similar to those of RFA, but lower rates of LTR and higher rates of CTA in large tumors compared with RFA were reported (*P* < 0.05). CTA rates were lower in patients treated with PEI (odds ratio [OR] 0.16, 95% confidence interval [CI] 0.06–0.42), and higher in those treated with PEI plus RFA (OR 2.28, 95% CI 1.19–3.60), with an increased incidence of fever (*P* < 0.05). LSA resulted in lower CTA rates (OR 0.32, 95% CI 0.13–0.81) and OS (hazard ratio 1.47, 95% CI 1.01–2.15), with a lower incidence of complications.

**Conclusions:**

Compared with RFA, identical effects were found in MWA and CRA groups. Fewer complications were observed in PEI and LSA group. PEI plus RFA appeared more effective, with a higher rate of complications. Well-designed randomized controlled trials are further needed to confirm above results.

## Background

Hepatocellular carcinoma (HCC) is the most common primary malignant tumor of the liver, with a poor prognosis. Currently, nearly 78% of cases occur in Asia, and the global incidence of HCC is increasing steadily [1, 2]. Although hepatic resection was previously recommended as the first-line choice for radical treatment, only about 15–20% of patients were deemed surgical candidates at the time of HCC diagnosis [3]. The application of liver transplantation is also limited, given the shortage of appropriate donors.

In the past 20 years, with the development of imaging techniques, such as ultrasonographic guidance, percutaneous ablation has become an important alternative treatment for small HCC and cases deemed unresectable by surgery [3]. Many different modalities have been proposed and accepted for ablation procedures; these include radiofrequency ablation (RFA), microwave ablation (MWA), percutaneous ethanol injection (PEI), laser ablation (LSA), cryoablation (CRA), high-intensity focused ultrasound (HIFU), and combinations thereof [4–7]. Ablative techniques result in the necrotization of tumor tissue by various mechanisms, such as thermal coagulation, rapid freezing, and chemical cell dehydration [4–7], with different post-ablative effects.

PEI was among the first ablative treatments for HCC; RFA has been recently employed and widely accepted for early-stage and unresectable HCC [3]. International guidelines for HCC management refer to these two methods [8]. However, other available ablative techniques have been reported to achieve effective therapeutic response, with subtle differences among them [6, 7]. Thus, clinicians planning HCC treatment by local ablation must select from the various ablative options. The literature comparing the effects of various ablative methods is limited, and the available data should be summarized and clarified.

The aim of the present systematic review and meta-analysis was to compare the outcomes of various ablation methods in HCC treatment, with summarization of the evidence supporting the selection of RFA and other ablative techniques. The rates of complete tumor ablation (CTA), local tumor recurrence (LTR), overall survival (OS), and complications of RFA were assessed relative to those of other percutaneous ablative techniques.

## Methods

### Search strategy and selection criteria

A computerized literature review was conducted to identify articles published between January 1995 and September 2016 using the PubMed, Embase, Cochrane Library, and China Biology Medicine databases. The initial year of 1995 was chosen because the first report on the use of RFA on a patient’s liver was published in this year. The search terms used were: “carcinoma, hepatocellular” (MeSH term) OR “hepatocellular carcinoma” (text) AND “radiofrequency ablation” (text) OR “high-intensity focused ultrasound ablation” (MeSH term) OR “high-intensity focused ultrasound” (text) OR “laser coagulation” (MeSH term) OR “laser ablation” (text) OR “microwave ablation” (text) OR “percutaneous ethanol injection” (text) OR “cryosurgery” (MeSH term) OR “cryoablation” (text) OR “ablation techniques” (MeSH term). The reference list of identified publications and review articles were checked manually to identify additional related articles. The last search was performed in September 2015.

The studies meeting all the following criteria were included: (1) full text available in English or Chinese, due to language limit; (2) the results providing data relative to CTA, LTR, OS, or complications, and compared between the outcome of RFA with that of other percutaneously ablative techniques on HCCs, such as MWA, PEI, LSA, CRA, HIFU, or a combination; (3) randomized controlled clinical trials; (4) high quality cohort studies after assessed by Newcastle-Ottawa scale (NOS).

The following forms of publications were excluded: (1) literature in the form of case reports, editorials, reviews, and conference abstracts; (2) the full text not written in English or Chinese; (3) lack of the required postoperative data in the results, and no response after attempts to connect with the author; (4) duplicate data if recent studies were already included; (5) the animal or in vitro research; (6) ablative techniques combined with transhepatic artery chemoembolism; (7) ablation under laparoscope or during surgical operation.

### Quality assessment and data extraction

NOS and Cochrane collaboration’s tool for bias risk assessment were used for quality assessment of cohort studies and RCTs, respectively, by two reviewers (L.LW, Y.M). Different opinions were solved through consultation.

For observational cohort studies, assessment of quality was performed in eight items described in Table 2. Two stars for comparability was acquired and one for the other items if the condition were met. A high-quality study was defined as a study with seven or more stars in total.

For RCTs, low, unclear or high risk of bias in the items including random sequence generation, allocation concealment, blinding of outcome assessment, incomplete outcome data, and selective reporting, was assessed for each study.

Two of the authors (L.W, Zh.YF) independently extracted data, including: (1) country and publication year; (2) number of patients; (3) age; (4) number and size of the tumor; (5) number of patients in CTA; (6) rate of LTR; (7) data about OS or from the OS curve; (8) number of patients with complications. Engauge Digitizer 4.1 was used to extract survival data from OS curves.

CTA was defined as no enhancement in ablated areas on enhanced CT, MRI, or US within 4 weeks after ablation, while LTR was considered as the tumor enhancement near the ablated areas on enhanced CT or MRI during follow-up period. Major complications were mentioned as the events leading to substantial morbidity and disability increase the duration of stay in hospitals or the caring rank, such as subcapsular/intrahepatic hematoma, biliary system injury, hemathorax, liver infarction, liver compensation, symptoms of breath holding and incomplete intestinal obstruction. The other complications were considered minor, such as pain, fever, skin burns, pleural effusion, liver transaminase change, and tumor seeding.

### Statistical analysis

The comparison of MWA versus RFA, PEI versus RFA, PEI plus RFA versus RFA, CRA versus RFA, LA versus RFA, and HIFU versus RFA were conducted. Observational studies and RCTs were conducted through meta-analysis, respectively. Reviewer Manager (RevMan; computer program; version 5.2; The Nordic Cochrane Center, The Cochrane Collaboration, 2012, Copenhagen, Denmark) was used for pooling data.

In the meta-analysis, hazard ratio (HR) with 95% CI was calculated for comparison of survival data. Results about CTA, LTR, and complications were compared by calculating odds ratio (OR) with 95% CI. Peto OR was used for the low incidence of events. Heterogeneity was assessed by calculating *I* ^2^. *I* ^2^ > 50% was considered as exist of significant heterogeneity. Fixed-effect model was conducted in statistical analyses if there was no obvious heterogeneity; otherwise the random-effect model was used. Inverse variance was used for evaluation of continuous variables, such as the diameter of tumor. Publication bias was evaluated by the funnel plot and Egger’s regression. In all analyses, *P* < 0.05 was regarded as statistically significant.

## Results

### Characteristics of included studies

**Fig. 1:**
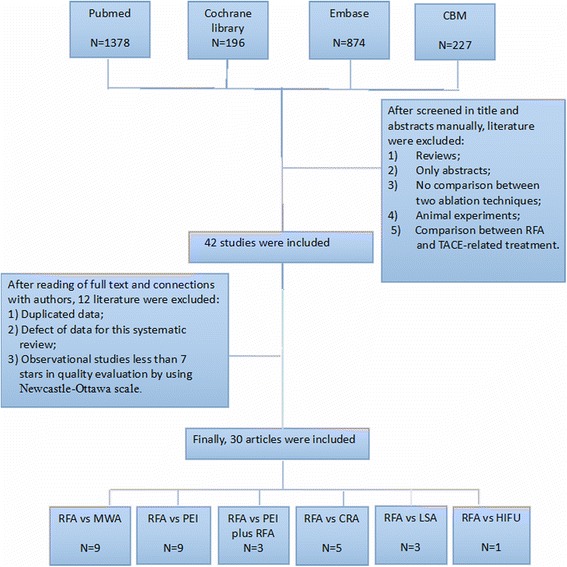
Flowchart of articles search

The initial database search yielded 2675 publications (Fig. 1), 2633 of which were excluded upon the screening of titles and abstracts by two reviewers (Zh.XD, H.GB). The full texts of the remaining 42 studies were assessed, and those with duplicate data, lack of assessment of the required outcome, and/or poor quality were excluded. The final sample comprised 30 articles (14 cohort studies, 16 randomized controlled trials [RCTs]). The cohort studies were published between 1999 and 2015, and the RCTs were published between 2002 and 2015. Table 1 shows the main characteristics of included studies [9–36].

**Table 1:** Characteristics of studies comparing the results of radiofrquency ablation with those of other local ablations on HCCs

First author	Country/year	Type	Arms	NP	Age (years)^c^	NL	Size, mean (range, cm)	CTA (n1/n0)	LTR	Survival rates (%)	CM (%)
Vogl TJ [9]	Egypt/2015	Cohort	MWA	28	60 (45–68)	36	3.6 ± 1.24^d^	32/36	1 year:2.8% (1/36)^a^	1 year:100; 2 years:96; 3 years:79	NR
			RFA	25	57 (40–64)	32	3.2 ± 1.17	27/32	1 year:3.1% (1/32)^a^	1 year:100; 2 years:88; 3 years:72	NR
Zhang L [10]	China/2013	Cohort	MWA	77	54 (26–76)	105	NR	91/105	10.5% (11/105) ^a^	1 year:92.2; 3 years:51.7; 5 years:38.5	2.6^e^
			RFA	78	54 (30–80)	97	NR	78/93	11.8% (11/93) ^a^	1 year:91.0; 3 years:64.1; 5 years:41.3	2.6^e^
Lu MD [11]	China/2005	Cohort	MWA	49	50.1 (24–74)	98	2.5 ± 1.2	93/98	11.8% (11/93)^a^	1 year:81.6; 2 years:61.2; 3 years:50.5; 4 years:36.8	8.2^e^
			RFA	53	54.5 (20–74)	72	2.6 ± 1.2	67/72	20.9% (14/67)^a^	1 year:71.7; 2 years:47.2; 3 years:37.6; 4 years:24.2	5.7^e^
Ding J [12]	China/2013	Cohort	MWA	113	59 (30–86)	131	2.55 ± 0.89	129/131	10.9% (14/129)^a^	1 year:98.0; 2 years:90.7; 3 years:77.6; 4 years:77.6	27^e^
			RFA	85	58 (40–77)	98	2.38 ± 0.81	97/98	5.2% (5/97)^a^	1 year:98.7; 2 years:92.3; 3 years:82.7; 4 years:77.8	2.4^e^
Zhang NN [13]	China/2014	Cohort	MWA	45	59 (37–73)	60	NR	58/60	2 years:40% (18/45)	1 year:95.6; 2 years:86.7	NR
			RFA	56	57 (28–77)	68	NR	58/68	2 years:42.9% (26/56)	1 year:94.6; 2 years:89.3	NR
Ohmoto K [14]	Japan/2008	Cohort	MWA	49	64 (38–75)	56	1.7 ± 0.39^d^	56/56	1 year:13%; 2 years:16%; 3 years:19%; 4 years:19%	1 year:89; 2 years:70; 3 years:49; 4 years:39	NR
			RFA	34	67 (44–78)	37	1.6 ± 0.4	37/37	1 year:9%; 2 years:9%; 3 years:9%; 4 years:9%	1 year:100; 2 years:83; 3 years:70; 4 years:70	NR
Abdelaziz A [15]	Egypt/2014	RCT	MWA	66	56.8 ± 7.3	76	2.95 ± 1.03	73/76	3.9%	1 year:96.4; 2 years:62	3.0^e^
			RFA	45	53.6 ± 5	52	2.9 ± 0.97	49/52	13.5%	1 year:67.6; 2 years:47.4	11.1^e^
Shibata T [16]	Japan/2002	RCT	MWA	36	62.5 (52–74)	46	2.3 ± 0.78^d^	41/46	1 year:10% 2 years:24%	NR	11^e^
			RFA	36	63.6 (44–83)	48	2.2 ± 0.32	46/48	1 year:4% 2 years:12%	NR	3^e^
Tian WS [17]	China/2014	RCT	MWA	60	55.3 (21–74)	79	2.6 ± 1.3	73/79	NR	NR	NR
			RFA	60	55.3 (21–74)	86	2.2 ± 0.9	77/86	NR	NR	NR
Morimoto M [18]	Japan/2007	Cohort	PEI	43	69 ± 7		NR^g^	35/43^b^	58% (25/43)	NR	NR
			RFA	110	68 ± 7		NR	90/110^b^	53% (58/110)	NR	NR
Seror O [19]	France/2006	Cohort	PEI	57	62(42–76)	72	2.5 ± 0.5	69/72	29.8% (17/57)	1 year:89.4; 2 years:70.8	6.9
			RFA	60	62(42–76)	72	2.5 ± 0.5	71/72	18.3% (11/60)	1 year:96.6; 2 years: 91.2	15
Wakui N [20]	Japan/2010	Cohort	PEI	13	70.3(50–83)	15	1.06 ± 0.27	15/15	1 year:9%	NR	NR
			RFA	10	69.3(65–75)	10	1.11 ± 0.27	10/10	1 year:44%	NR	NR
Luo BM [21]	China/2005	Cohort	PEI	71	31–72	85	2.21 ± 1.48	66/85	NR	1 year:80.0; 2 years:60.4; 3 years:52.5; 5 years:33.3	NR
			RFA	118	31–72	153	2.39 ± 1.57	141/153	NR	1 year:94.6; 2 years:73.2; 3 years:63.5	NR
Giorgio A [22]	Italy/2011	RCT	PEI	143	72(68–79)	143	2.27 ± 0.48	NR	12.6% (18/143)	1 year:95; 2 years:83; 3 years:78; 4 years:70; 5 years:68	1.9
			RFA	128	70(68–74)	128	2.34 ± 0.45	NR	11.7% (15/128)	1 year:95; 2 years:90 ;3 years:83; 4 years:73; 5 years:70	0.9
Brunello F [23]	Italy/2008	RCT	PEI	69	70.3	88	2.25 ± 0.54	46/69^b^	44/69 (63.8%)	1 year:85.5; 2 years:58.0; 3 years:24.6; 4 years:7.2	17.4
			RFA	70	69.0	89	2.42 ± 0.49	66/70^b^	34.3% (34/70)	1 year:94.3; 2 years:58.6; 3 years:25.7; 4 years:10	14.3
Lin SM [24]	Taiwan/2004	RCT	PEI	52	59 ± 10	69	2.90 ± 0.80	46/52^b^	1 year:23% 2 years:45% 3 years:45%	1 year:85; 2 years:61; 3 years:50	NR
			RFA	52	62 ± 11	67	2.8 ± 0.8	50/52^b^	1 year:12% 2 years:18% 3 years:18%	1 year:90; 2 years:82; 3 years:74	NR
Shiina S [25]	Japan/2005	RCT	PEI	114	NR	192	NR	192/192	11.4% (13/114)	1 year:95; 2 years:82; 3 years:65; 4 years:57	2.6
			RFA	118	NR	187	NR	187/187	1.7% (2/118)	1 year:97; 2 years:92; 3 years:82; 4 years:74	5.1
Lencioni RA [26]	Italy/2003	RCT	PEI	50	69(40–82)	73	2.8 ± 0.8	60/73	26% (13/50)	1 year:96; 2 years:88	NR
			RFA	52	67(52–78)	69	2.8 ± 0.6	63/69	5.8% (3/52)	1 year:100; 2 years:98	NR
Azab M [27]	Egypt/2011	RCT	PEI + RFA	30	46–77	33	NR	29/33	1 year:6% 1.5 years:6%	1 year:96.7; 1.5 years:86.7	NR
			RFA	30	46–77	33	NR	16/30	1 year:9% 1.5 years:12%	1 year:90; 1.5 years:76.7	NR
			PEI	30	46–77	32	NR	L 75%	1 year:15% 1.5 years:21%	1 year:83.3; 1.5 years:86.7	NR
Wong SN [28]	Taiwan/2008	Cohort	PEI + RFA	33	66.4 ± 9.7	50	2.8 ± 1cm	44/50	1 year:24% 2 years:60%	NR	11.5^f^
			RFA	85	66.4 ± 9.7	114	2.5 ± 0.1cm	92/114	1 year:21% 2 years:30%	NR	19.4^f^
Zhang YJ [29]	China/2007	RCT	PEI + RFA	66	53.3 ± 11.3	107	NR	52/66^b^	34.8% (23/66)	1 year:95.4; 2 years:89.2; 3 years:75.8; 4 years:63.3;	46
			RFA	67	52.2 ± 10.3	103	NR	48/67^b^	49.2% (33/67)	1 year:89.6; 2 years:68.7; 3 years:58.4; 4 years:50.3	30
Pearson AS [30]	USA/1999	Cohort	CRA	54	NR	88	3.6(0.8–9.0)^d^	NR	13.6% (12/54)	NR	40.7
			RFA	92	NR	138	3.8(0.5–12.0)	NR	3.3% (3/92)	NR	3.3
Adam R [31]	France/2002	Cohort	CRA	15	60.1 ± 9.6	20	2.22 ± 1.05	16/20	45.5% (5/11)	1 year:66	29
			RFA	17	63.5 ± 9.9	21	2.80 ± 1.67	18/21	14.3% (2/14)	1 year:61	24
Dunne RM [32]	USA/2014	Cohort	CRA	25	67.8 ± 10.7	39	2.8(1.5–4.9)^d^	38/39	13.5% (5/37)^a^	NR	39.4^f^
			RFA	22	64.4 ± 10.8	39	2.0(0.4–6.3)	36/39	21.4% (6//28)^a^	NR	26.7^f^
Ei S [33]	Japan/2015	Cohort	CRA	55	69 (65-74)	NR	2.5 (2.0-3.0)^d^	100%	2 years:38% (21/55)	2 years:≦2 cm:88%; >2 cm:86%	10.9
			RFA/MWA	64	69 (64–74)	NR	1.9 (1.5–2.3)	100%	2 years:34% (22/64)	2Y:≦2 cm:95%; >2 cm:85%	10.9
Wang CP [6]	China/2015	RCT	CRA	180	53.87 ± 9.587	199	NR	196/199	1 year:3%; 2 years:7% 3 years:7% 10/180	1 year:97; 3 years:67; 5 years:40	3.9
			RFA	180	53.34 ± 8.905	189	NR	181/189	1 year:9% 2 years:11% 3 years:11% 18/180	1 year:97; 3 years:66; 5 years:38	3.3
Di Cos GG [34]	Italy/2013	RCT	LSA	70	70 (36–84)	80	2.62 ± 1.04	77/80	22.9% (16/70)	1 year:94; 3 years:80	NR
			RFA	70	70 (50–83)	77	2.55 ± 0.66	75/77	25.7% (18/70)	1 year:94; 3 years:89	NR
Ferrari FS [35]	Italy/2007	RCT	LSA	41	68.27 (51–82)	45	2.89 ± 0.73	35/45	19.5% (8/41)	1 year:88.6; 2 years:70.4; 3 years:56.6; 4 years:40.2	0
			RFA	40	70.53 (59–80)	50	2.67 ± 0.81	47/50	17.5% (7/40)	1 year:92.2; 2 years:75.0; 3 years:61.3; 4 years:54.6	0
Orlacchio A [7]	Italy/2014	RCT	LSA	15	73. 50 ± 6.70	15	2.34 ± 0.82	10/15	1 year:40% (6/15)	1 year:100	13.3
			RFA	15	71.50 ± 4.60	15	2.41 ± 0.71	13/15	1 year:13.3% (2/15)	1 year:100	53.3
Chan AC [36]	China/2013	Cohort	HIFU	27	63 (44–75)	NR	1.7(0.9–5.0)	23/27	NR	1 year:96.3; 2 years:81.5; 3 years:69.8	7.4^f^
			RFA	76	62 (28–84)	NR	1.8(0.7–4.9)	65/76	NR	1 year:92.1; 2 years:76.1; 3 years:64.2	22.4^f^

*NP* number of patients, *NL* number of lesions, *n1* number of lesions with complete necrosis, *n0* number of lesions undergoing ablation, *CM* patient-related complications, *NR* no record, *Y* years.

^a^Lesion-related local recurrence

^b^Number of patients with complete necrosis/number of patients undergoing ablation

^c^Age recorded with mean or median (range, year) or mean ± standard deviation

^d^Median size (range)

^e^Major complications

^f^Procedure-related

### Quality of included studies

**Fig. 2:**
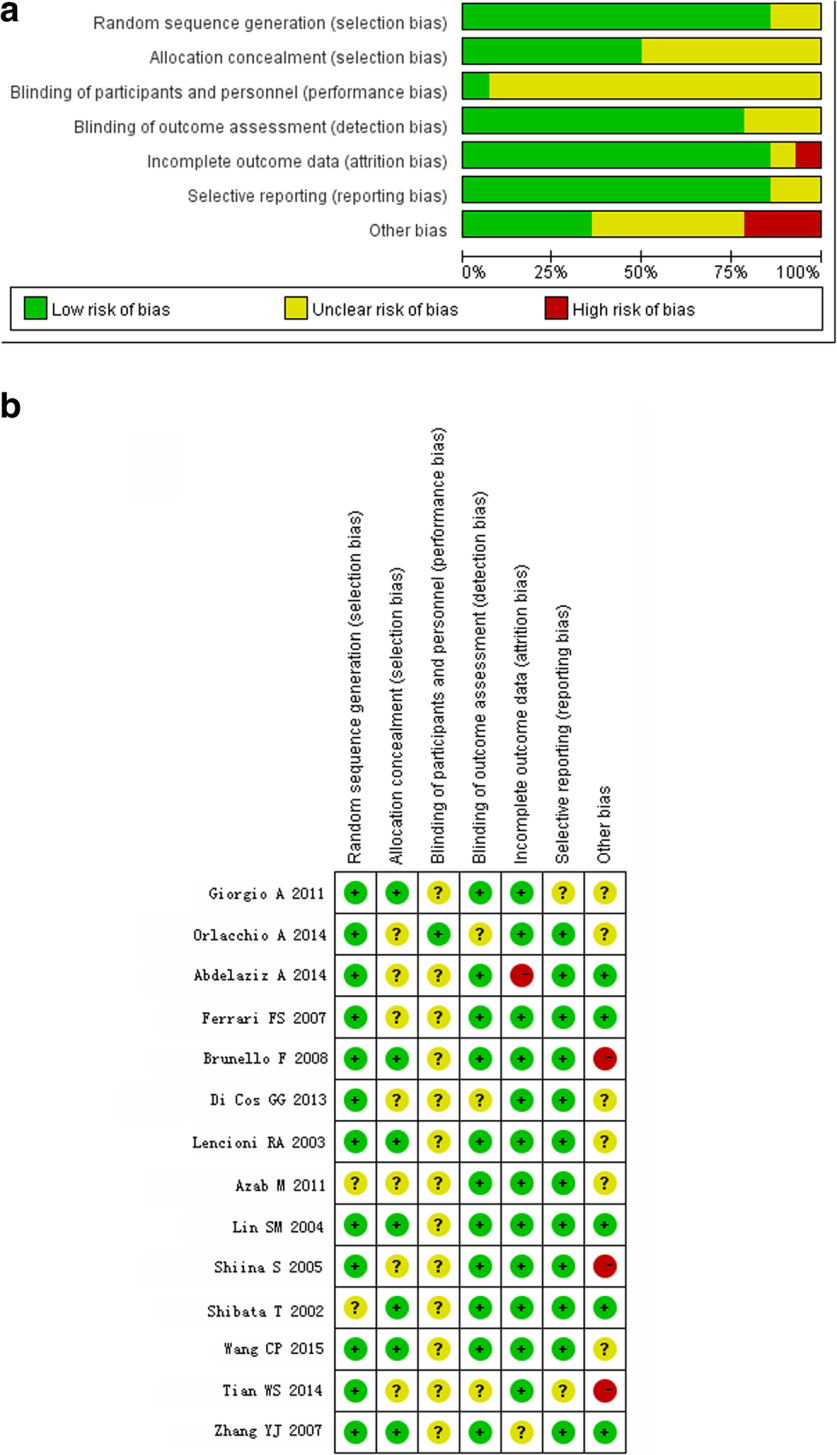
Methodological quality summary of randomized controlled trials. **a** Risk of bias graph. **b** Risk of bias summary

According to the Cochrane Collaboration tool, 13 of the RCTs were affected by performance bias (Fig. 2). Because the ablative techniques require the use of different equipment throughout the duration of therapy, blinding of physicians was difficult. Whether the outcome was assessed blindly was not indicated in any trial, but the assessment of computed tomography (CT) and magnetic resonance imaging (MRI) data was objective. Therefore, no obvious detection bias in outcome evaluation was identified. Factors such as the use of “one-shot” PEI [23], the inclusion of small (diameter < 3 cm) tumors [25], and short follow-up periods [17] resulted in the high risk of other biases.

The 16 cohort studies were given 7–9 stars upon NOS scale evaluation (Table 2). One of these studies was from Africa, 2 were from Europe, 11 were from Asia, and 2 were from the United States of America.

**Table 2:** Methodological quality assessment using the Newcastle-Ottawa scale for the cohort studies

First author	Arms	Representativeness of exposed cohort	Selection of the non-exposed cohort	Ascertainment of exposure	Demonstration that outcome of interest wars not present at start	Comparability of cohorts on the basis of the design	Assessment of outcome	Was follow-up long enough for outcomes to occur	Adequacy of follow-up of cohort
Lu MD [11]	MWA vs. RFA	*	*	*	*	**	*	*	*
Ohmoto K [14]	MWA vs. RFA	*	*	*	*	**	*	*	*
Zhang L [10]	MWA vs. RFA	*	*	*	*	*	*	*	*
Ding J [12]	MWA vs. RFA	*	*	*	*	**	*	*	*
Vogl TJ [9]	MWA vs. RFA	*	*	*	*	**		*	*
Zhang NN [13]	MWA vs. RFA	*	*	*	*	**	*	*	*
Wakui N [20]	PEI vs. RFA	*	*	*	*	**	*	*	*
Morimoto M [18]	PEI vs. RFA	*	*	*	*	**	*	*	*
Luo BM [21]	PEI vs. RFA	*	*	*	*		*	*	*
Seror O [19]	PEI vs. RFA	*		*	*	**	*	*	*
Adam R [31]	CRA vs. RFA	*	*	*	*	*	*	*	*
Pearson AS [30]	CRA vs. RFA	*	*	*	*	**	*	*	*
Ei S [33]	CRA vs. RFA	*	*	*	*	*	*	*	*
Dunne RM [32]	CRA vs. RFA	*	*	*	*	**	*	*	*
Wong SN [28]	PEI + RFA vs. RFA	*	*	*	*	*	*	*	*
Chan AC [36]	HIFU vs. RFA	*	*	*	*	*	*	*	*

A study can be awarded a maximum of * for each item

A maximum of ** can be given for Comparability

### MWA versus RFA

Six cohort studies [9–14] and three RCTs [15–17] were included in the comparison of the effects of MWA and RFA. The diameters of tumors treated with MWA and RFA were similar in cohort studies (mean difference 0.11, 95% confidence interval [CI] −0.01 to 0.23) [9, 11, 12, 14] and RCTs (mean difference 0.16, 95% CI −0.01 to 0.34) [15–17]. Except for studies without records, number of cases with single HCC showed identical in three cohort studies (OR 0.66, 95% CI 0.40–1.08) [9, 10, 13] and 2 RCTs (OR 1.24, 95% CI 0.58–2.65) [15, 16]. The baseline background of patients including age, Child-Pugh class, tumor marker, and tumor location were homogeneous [10–17].

**Fig. 3:**
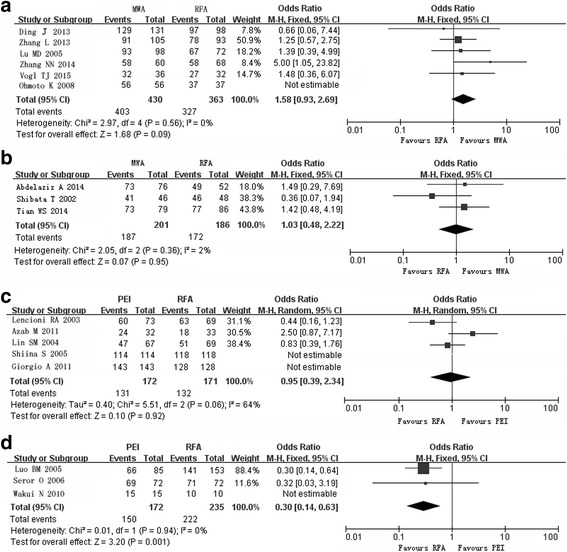
Forest plot of pooled rates of completed tumor ablation after radiofrequency ablation (*RFA*) and other techniques. **a** Microwave ablation (*MWA*) versus RFA in cohort studies. **b** MWA versus RFA in randomized studies. **c** Percutaneous ethanol injection (*PEI*) versus RFA in randomized studies. **d** PEI versus RFA in cohort studies

No significant difference in the CTA rate, OS, 1- or 3-year survival rate was detected between the MWA and RFA groups (Fig. 3, Tables 3 and 4). In the cohort studies, LTR was reported in 15.2% (60 of 394) of patients in the RFA group and 13.8% (66 of 479) of patients in the MWA group. No significant difference in LTR was detected between the MWA and RFA groups in the fixed-effect model (Table 3).

**Table 3:** Meta-analysis of effects of microwave ablation versus radiofrequency ablation on HCCs

	CTA	1-year SR	3-year SR	OS	LTR	Major complications
Cohort studies	MWA 86.7–100%	MWA 81.6–98%	MWA 49–79%	HR 0.80	OR 0.95	OR 1.23
	RFA 83.9–100%	RFA 67.6–98.7%	RFA 37.6–82.7%	95% CI 0.62–1.04	95% CI 0.64–1.41	95% CI 0.45–3.37
	*P* > 0.05	*P* > 0.05	*P* > 0.05			
	[9–14]	[9–14]	[9–14]	[9–14]	[9–14]	[10–12]
RCT	MWA 89.1–96.1%	NA	NA	HR 0.58	OR 1.19	OR 0.80
	RFA 89.5–95.8%			95% CI 0.22–1.56	95% CI 0.20–7.06	95% CI 0.26–2.49
	*P* > 0.05					
	[15–17]			[15, 17]	[15–17]	[15, 16]

*CTA* complete tumor ablation, *SR* survival rates, *OS* overall survival, *LTR* local tumor recurrence, *MWA* microwave ablation, *RFA* radiofrequency ablation, *HR* hazard ratio, *RCT* randomized controlled trial, *NA* not applicable. Those in *square brackets* were numbers of references

**Table 4:** Meta-analysis of overall survival rates of other ablative technique versus radiofrequency ablation

	Cohort studies	RCTs
MWA vs. RFA	HR 0.80 95% CI 0.62–1.04	HR 0.58 95% CI 0.22–1.56
	[9–14]	[15, 17]
PEI vs. RFA	HR 1.67 95% CI 1.16–2.40	HR 1.26 95% CI 0.96–1.66
	[19, 21]	[22–26]
PEI plus RFA vs. RFA	NA	HR 0.61 95% CI 0.36–1.02
		[27, 29]
LSA vs. RFA	NA	HR 1.47 95% CI 1.01–2.15
		[7, 34, 35]

*MWA* microwave ablation, *RFA* radiofrequency ablation, *PEI* percutaneous ethanol injection, *LSA* laser ablation, *HR* hazard ratio, *RCT* randomized controlled trial, *NA* not applicable. Those in *square brackets* were numbers of references

**Fig. 4:**
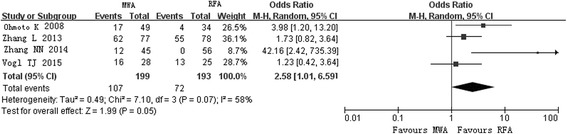
Forest plot of pooled rates of fever in the microwave ablation group and the radiofrequency ablation group

Major complications occurred in 4.39% (15 of 342) of patients in the MWA group and 4.39% (13 of 296) of patients in the RFA group (Table 3) [10–12, 15, 16]. Four studies referred to subcapsular or intrahepatic hematoma, which occurred in 2.2% (5 of 228) of patients in the MWA group and 0.9% (2 of 212) of patients in the RFA group [10, 11, 15, 16]. For hematoma, Peto ORs were 7.94 (95% CI 0.82–77.04) for the cohort studies and 0.80 (95% CI 0.14–4.55) for the RCTs. Pain was present in 62.8% (125 of 199) of patients in the MWA group and 45.1% (87 of 193) of patients in the RFA group (OR 1.82, 95% CI 0.75–4.39) in 4 cohort studies [9, 10, 13, 14]. One study from China reported the incident of a needle breaking in one case (13). The OR for pleural effusion in cohort studies was 1.52 (95% CI 0.84–2.75) [9, 10, 13, 14]. Fever occurred in 53.8% (107 of 199) of patients in the MWA group and 37.3% (72 of 193) of patients in the RFA group (*P* = 0.05) in the 4 cohort studies (Fig. 4).

Subgroup analysis of tumors with diameters >3.0 cm revealed no difference in CTA in 2 cohort studies [11, 13] and two RCTs [15, 16]; the fixed-effect model yielded ORs of 2.61 (95% CI 0.93–7.33) and 0.20 (95% CI 0.03–1.42), respectively. For LTR, the OR was 0.73 (95% CI 0.38–1.39) [11, 13]. Although no difference in OS was detected for tumors with diameters of 3.1–5.0 cm, Zhang et al. [10] reported better 1-, 3-, and 5-year disease-free survival rates in patients treated with RFA (74.2, 54.8, and 45.2%) than in those treated with MWA (53.3, 26.8, and 17.1%; *P* = 0.018) (10).

### PEI versus RFA

Ten studies (six RCTs [22–27] and four cohort studies [18–21]) were included in the investigation of the therapeutic effects of PEI compared with RFA. No significant difference in tumor diameter was detected between groups in the cohort studies (mean difference –0.03, 95% CI −0.16 to 0.09) or RCTs (mean difference −0.07, 95% CI −0.15 to 0.01) in a fixed model. Assessed in two cohort studies, number of single tumor in PEI group was smaller than that in RFA group (OR 0.40, 95% CI 0.23–0.70) [18, 19], while no significant difference was detected in five RCTs (OR 0.89, 95% CI 0.64–1.24). Age, Child-Pugh class, and tumor marker were described identical between different groups in all the studies.

CTA rates ranged from 70.1 to 100%, and were lower in the PEI group than in the RFA group (Fig. 3). For 3 of RCTs [24, 26, 27], a fixed-effect model yielded an OR for lesion-related CTA in the PEI group compared with that in the RFA group of 0.16 (95% CI 0.06–0.42) (Fig. 3c). For 3 of the cohort studies, the fixed-effect model yielded an OR of 0.30 (95% CI 0.14–0.63) for lesion-related CTA; the fourth cohort study [18] was not included in this model because patient-related CTA rates were reported (Fig. 3d).

A random-effect model comparing the PEI and RFA groups yielded ORs for LTR of 3.37 (95% CI 1.00–11.32) for RCTs [24–26] and 1.41 (95% CI 0.83–2.40) for cohort studies [18–20]. The 1- and 3-year LTR rates were higher in the PEI group than in the RFA group (OR 2.25, 95% CI 1.15–4.83 and OR 2.44, 95% CI 1.10–5.41, respectively) [22, 24, 25].

In the meta-analysis comparing OS in the PEI and RFA groups, no significant difference was detected for the RCTs (Table 4) [22–26]. No difference in the number of patients experiencing complications was detected between groups (Peto OR 0.90, 95% CI 0.47–1.73) [22–25]. Serious adverse events described in the RCTs included neoplasm seeding (three cases), transient jaundice, skin burn, hepatic infarction, hemoperitoneum, and right hemothorax (one case each) in the RFA group, and neoplasm seeding (two cases), liver abscess, hemoperitoneum, and portal vein thrombosis (one case each) in the PEI group. The comparison performed in one cohort study showed no difference in the incidence of complications between groups [19].

Brunello et al. [23] reported a higher rate of CTA in the RFA group (68.1%) than in the PEI group (28.3%) for tumors with diameters >20 mm (*P* < 0.05) [23]. For tumors with diameters ≥20 mm and ≥ 30 mm, Lin [24] reported lower 1-, 2-, and 3-year LTR rates in the RFA group than in the PEI group (11, 18, and 18% vs. 18, 37, and 37% [*P* < 0.05] and 13, 24, and 24% vs. 31, 52, and 52% [*P* < 0.05], respectively). One-, 2-, and 3-year OS rates were higher in the RFA group (87, 73, and 62%) than in the PEI group (82, 55, and 36%; *P* < 0.05) [24].

### PEI plus RFA versus RFA

Two RCTs [27, 29] and one cohort study [28] were included in this assessment. All the three studies showed no significant difference in tumor diameter, number, liver function, and tumor markers between the PEI plus RFA and RFA groups [27].

**Fig. 5:**
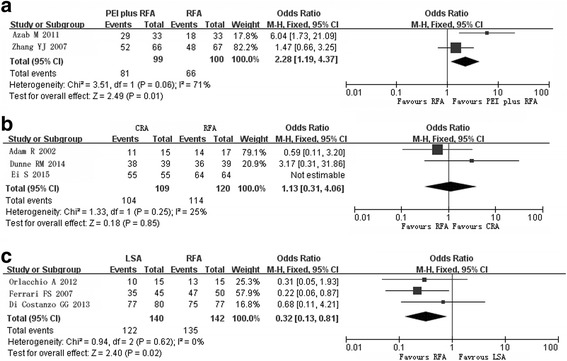
Forest plot of pooled rates of completed tumor ablation after radiofrequency ablation (*RFA*) and othertechniques. **a** Percutaneous ethanol injection plus RFA versus RFA. **b** Cryoablation versus RFA **c** Laser ablation versus RFA

In a meta-analysis of data from the two RCTs, the fixed-effect model showed a significantly higher rate of CTA in the PEI plus RFA group than in the RFA group (OR 2.28, 95% CI 1.19–3.60; Fig. 5a). A fixed-effect model yielded an OR of 0.54 (95% CI 0.28–1.03, *P* = 0.06) for LTR in the PEI plus RFA group compared with the RFA group. OS rates did not differ between groups (Table 4).

The incidence of fever was higher in the PEI plus RFA group than in the RFA group (Peto OR 2.22, 95% CI 1.20–4.09), whereas the incidence of pain did not differ significantly (OR 1.36, 95% CI 0.74–2.96).

Tumor diameter ≤3 cm was reported as a significant predictor of higher CTA in two studies [27, 28]. For tumors with diameters of 3.1–5 cm, Zhang et al. [29] reported a higher OS rate in the PEI plus RFA group (77.6%) than in the RFA group (48.2%) (*P* < 0.05).

### CRA versus RFA

Four cohort studies [30–33] and one RCT [6] were included in the comparison of the effects of CRA and RFA. In all the studies, it was described that CRA and RFA group did not differ in tumor number and serum laboratory tests.

The cohort studies reported on CTA after CRA and RFA (fixed-effect model: OR 1.13, 95% CI 0.31–4.06; Fig. 5b); CTA rates ranged from 73.3 to 100% in the CRA group and from 82.4 to 100% in the RFA group. The RCT reported CTA rates near 98.3% in the CRA group and 95.6% in the RFA group (*P* > 0.05).

In the cohort studies, LTR occurred in 27.4% (43 of 157) patients in the CRA group and 16.7% (33 of 198) patients in the RFA group (random-effect model: OR 2.10, 95% CI 0.65–6.78). In the RCT, the incidence of LTR was 5.6% in the CRA group and 10% in the RFA group (OR 0.53, 95% CI 0.24–1.18). Only two studies supplied OS data [6, 31]. Wang et al. [6] reported 1-, 3- and 5-year OS rates of 97, 67, and 40%, respectively for CRA, and similar data for RFA (*P* > 0.05).

The incidence of patient-related complications ranged from 3.8 to 40.7% in the CRA group and from 3.3 to 24% in the RFA group [18–20]. A random-effect model yielded an OR of 5.02 (95% CI 0.33–77.07) for data on the incidence of complications from two cohort studies [18, 20]. In the RCT, 7 of 180 patients in the CRA group and 6 of 180 patients in the RFA group had complications (OR 1.17, 95% CI 0.39–3.56). Various complications were mentioned in these studies; the most common were fever (154 of 195 patients in the CRA group and 145 of 197 patients in the RFA group) [6, 31], pain (44 of 180 patients in the CRA group and 98 of 180 patients in the RFA group), and abscess (12 of 234 patients in the CRA group and 4 of 272 in the RFA group) [6, 30]. Pleural effusion occurred in 10 patients treated with CRA and 2 patients receiving RFA [16, 30, 33]. In one study [32], thrombocytopenia occurred in 4 of 25 patients in the CRA group and 1 of 22 patients in the RFA group, and myoglobinemia occurred in 3 of 25 patients following CRA and in no patient after RFA.

A lower LTR rate in the CRA group than in the RFA/MWA group for tumors with diameters >2 cm (*P* = 0.006) [33]. Although tumors were larger in the CRA group in that study, the rates of complications did not differ significantly.

### LSA versus RFA

Three RCTs were included in this assessment [7, 34, 35]. A fixed-effect model revealed no significant difference in tumor diameter (mean difference 0.11, 95% CI −0.08 to 0.30) between the LSA and RFA groups. And number of single lesion did not differ between two groups (OR 1.38, 95% CI 0.64–2.98) [34, 35], while no record was detected in another RCT [7]. Treatment groups were homogeneous with regard to laboratory findings in three RCTs.

CTA rates ranged from 66.7 to 96.2% in the LSA group and from 86.7 to 97.4% in the RFA group (OR 0.32, 95% CI 0.13–0.81; Fig. 5c). LTR occurred in 30 of 126 patients after LSA and in 27 of 125 patients after RFA (OR 1.70, 95% CI 0.67–4.30). OS was better in the RFA group than in the LSA group (Table 4). Ferrari FS revealed that age, gender, and tumor marker did not affect survival, but in Child-Pugh class A group better survival rates were acquired after RFA than those after LSA. The same did not apply to class B cases [35].

The Peto OR for the incidence of complications was 0.57 (95% CI 0.31–1.07). Ferrari et al. [35] reported no complication or neoplastic seeding. Orlacchio et al. [7] reported minor complications occurring in 2 of 15 patients receiving LSA and 8 of 15 patients receiving RFA, including pleural effusion in 1 LSA case and 4 RFA cases, perihepatic effusion in 1 LSA case and 3 RFA cases, and subcapsular hematoma in 1 RFA case. Di Costanzo et al. [34] recorded moderate pain in 33% of patients receiving LSA and 36% of those receiving RFA, and self-limiting fever in 35% of patients in each group (*P* > 0.05).

### HIFU versus RFA

Only one cohort study referring to HIFU versus RFA was included [36]. No obvious different therapeutic effects were detected between two groups. As it was reported, OS rates were above 60% and CTA were more than 80% in both groups (*P* > 0.05). Procedure-related complications occurred in the RFA group comparable with HIFU group (*P* = 0.06) (Table 1).

### Publication bias

**Fig. 6:**
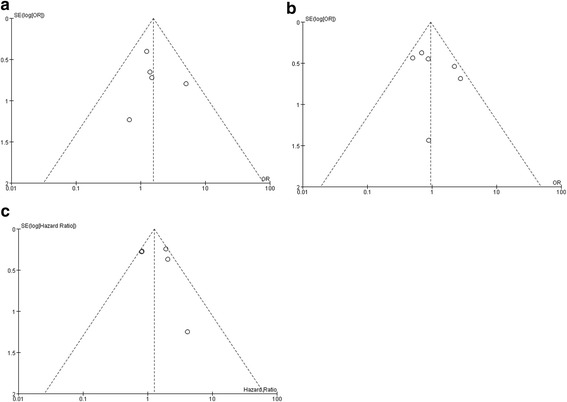
Funnel plot of pooled data. **a** completed tumor ablation between the microwave ablation (MWA) group and the radiofrequency ablation (RFA) group. **b** local tumor recurrence between the MWA group and the RFA group. **c** overall survival between the percutaneous ethanol injection group and the RFA group

Funnel plot and Egger’s test were used in the meta-analyses with more than five individual studies pooled in. No obvious asymmetry and *P* value over 0.05 were detected, which suggested there was no evidence of publication bias (Fig. 6).

## Discussion

Although great progress has been made in RFA, and this modality is considered to be important in HCC management, many physicians have used other ablative techniques in attempts to achieve better outcome. Currently, the selection of an ablation technique depends mainly on the doctor’s experience and the patient’s consent, as detailed indications for each modality have not been defined clearly. In the present study, we compared the therapeutic effects of multiple ablative techniques using data available in the literature to provide insight into modality selection.

MWA employs electromagnetic waves from electrodes to induce high temperatures in local areas, coagulating tissue; this principle is similar to that of RFA. In the current study, CTA rates exceeded 80% for MWA and RFA, OS and LTR rates were similar, and complication rates were similarly low. Only the incidence of fever was higher in the MWA group than in the RFA group, which may indicate that MWA was more invasive. In subgroups defined according to tumor size, no difference was detected between groups, but the results did indicate a possible trend toward an advantage of MWA for larger tumors.

As a non-thermal ablation, the use of PEI for the treatment of HCC was popular before the widespread use of RFA. In this study, we observed lower CTA rates and higher LTR rates in the PEI groups than in the RFA groups. The intratumor fibrous septum may interfere with the injection of ethanol [37]. Thus, the better outcome of RFA may be related to the greater predictability of ablation than in PEI. On the other side, few minor complications were described in PEI cases, whereas serious events, including skin burns, hepatic infarction, and hemothorax, occurred after RFA. The combined methods showed enhanced effects. A trend suggests that PEI-RFA may be utilized in 3.1–5-cm tumors. Kazutaka et al. [38] reported larger areas of coagulated necrosis in the PEI-RFA group (34 ± 29.3 cm^3^) than in the RFA group (6.5 ± 3.6 cm^3^; *P* < 0.0001). The amount of ethanol injected into the tumor was significantly and positively correlated with the volume of coagulated necrosis, but not with the energy requirement. Therefore, in the application of PEI-RFA in high-risk areas, such as near vessels or other important organs, ethanol injection helps to decrease the RFA energy, thereby protecting the surrounding tissues. However, in this study, a higher incidence of fever was found after PEI-RFA than after RFA.

CRA caused the tumor cell death through ice crystal formation during rapid freezing. We observed no obvious difference between CRA and RFA, while in two studies the tumor size in CRA was noted to be obviously larger than that in the RFA group [32, 33]. On the other hand, in a Japanese study [33], the tumor in CRA patients was located in close proximity to the hollow viscera such as the gallbladder, or important structure such as the hepatic hilum, and such location was not mentioned in the thermal ablation group. In addition, in the same study, lower LTR in the CRA group was reported than that in the RFA/MWA group for tumors with diameter greater than 2 cm. These outcome data may provide some information supporting that cyroablation may be more suited for larger HCCs or those in high risk areas, but more evidence is needed to make definitive conclusions. For complications, myoglobulinemia was not observed following RFA, but did occur in 3 of 33 procedures in CRA [32]. Thrombocytopenia and myoglobulinemia after CRA occurred in more procedures than that after RFA, but more data are needed for further statistical analysis [32, 33].

In the current study, it was shown that lower CTA rates and higher LTR rates were observed in the LSA patients, whereas higher OS rates in RFA patients were seen, particularly among larger HCCs (*P* < 0.05). The tendency of fewer complications was indeed detected in the LSA group. Thin needle for LSA may improve the ablative effects on the nodules with irregular shape or in high-risk location. In addition, in an Italian study, a lower cost of LSA was mentioned but had no statistical analysis [34].

There were some limitations in this study. Many studies have demonstrated that tumor size, number of single lesion, and Child-Pugh class were important prognosis factors [28, 35]. But in the above literature, data on these factors were not enough for meta-analysis in subgroup. Another limitation was that the device of techniques may have influence on its overall therapeutic effects. In the current study, we did not perform an analysis about the device, because the device varied greatly among different therapy centers. Finally, the number of papers included for CRA, LSA, and HIFU was small, especially in HIFU the meta analysis were not conducted and more evidence was needed in the future.

## Conclusions

Above all, the availability of multiple ablative techniques is the reason for optimism. It was pleasing that there have been many kinds of ablative techniques for treating HCCs. The combination of PEI and RFA apparently yielded a better prognosis than a single RFA. When the outcome of MWA appeared identical to that of RFA, a potential role of CRA used in larger tumors needs to be investigated. Less complications in PEI and LSA implied their application in high-risk areas for protecting the important organs. Indeed, well-designed randomized controlled trials are further needed to confirm the above results. With the different characteristics, multiple ablative techniques may be combined in one treatment procedure, so as to achieve better effects and avoid adverse events.

## Abbreviations

CRA: Cryoablation
CTA: Complete tumor ablation
HCC: Hepatocellular carcinoma
HIFU: High intensity focused ultrasound
HR: Hazard ratio
LSA: Laser ablation
LTR: Local tumor recurrence
MWA: Microwave ablation
OR: Odds ratio
OS: Overall survival
PEI: Percutaneous ethanol injection
RCT: Randomized controlled trial
RFA: Radiofrequency ablation
SR: Survival rates
